# β-carbolines that enhance GABA_A_ receptor response expressed in oligodendrocytes promote remyelination in an *in vivo* rat model of focal demyelination

**DOI:** 10.3389/fncel.2024.1369730

**Published:** 2024-04-17

**Authors:** Abraham Jotssel Cisneros-Mejorado, Rainald Pablo Ordaz, Edith Garay, Rogelio O. Arellano

**Affiliations:** Instituto de Neurobiología, Laboratorio de Neurofisiología Celular, Universidad Nacional Autónoma de México, Juriquilla, Mexico

**Keywords:** OPC, oligodendrocyte, diffusion tensor imaging, white matter, GABAergic signaling, β-carbolines, myelin

## Abstract

Demyelination is typically followed by a remyelination process through mature oligodendrocytes (OLs) differentiated from precursor cells (OPCs) recruited into the lesioned areas, however, this event usually results in uncompleted myelination. Potentiation of the remyelination process is an important target for designing effective therapeutic strategies against white matter loss. Here, it was evaluated the remyelinating effect of different β-carbolines that present differential allosteric modulation on the GABA_A_ receptor expressed in OLs. For this, we used a focalized demyelination model in the inferior cerebellar peduncle (*i.c.p.*) of rats (DRICP model), in which, demyelination by ethidium bromide (0.05%) stereotaxic injection was confirmed histologically by staining with Black-Gold II (BGII) and toluidine blue. In addition, a longitudinal analysis with diffusion-weighted magnetic resonance imaging (dMRI) was made by computing fractional anisotropy (FA), apparent diffusion coefficient (ADC) and diffusivity parameters to infer *i.c.p.* microstructural changes. First, dMRI analysis revealed FA decreases together with ADC and radial diffusivity (RD) increases after demyelination, which correlates with histological BGII observations. Then, we evaluated the effect produced by three allosteric GABA_A_ receptor modulators, the N-butyl-β-carboline-3-carboxylate (β-CCB), ethyl 9H-pyrido [3,4-b]indole-3-carboxylate (β-CCE), and 4-ethyl-6,7-dimethoxy-9H-pyrido [3,4-b]indole-3-carboxylic acid methyl ester (DMCM). The results indicated that daily systemic β-CCB (1 mg/Kg) or β-CCE (1 mg/Kg) administration for 2 weeks, but not DMCM (0.35 mg/Kg), in lesioned animals increased FA and decreased ADC or RD, suggesting myelination improvement. This was supported by BGII staining analysis that showed a recovery of myelin content. Also, it was quantified by immunohistochemistry both NG2^+^ and CC1^+^ cellular population in the different experimental sceneries. Data indicated that either β-CCB or β-CCE, but not DMCM, produced an increase in the population of CC1^+^ cells in the lesioned area. Finally, it was also calculated the *g*-ratio of myelinated axons and observed a similar value in those lesioned animals treated with β-CCB or β-CCE compared to controls. Thus, using the DRICP model, it was observed that either β-CCB or β-CCE, positive modulators of the GABA_A_ receptor in OLs, had a potent promyelinating effect.

## 1 Introduction

Central nervous system (CNS) myelination by oligodendrocytes (OLs) is an essential process for all brain functions in mammals (Knowles et al., 2022). Its importance is clear given the devastating effects of demyelinating diseases whose etiology has a wide range of factors and for which current therapeutic tools are generally only palliative. Demyelination of the nervous system causes significant impairments to vital sensory, motor, and cognitive functions. The origin of this condition may involve automimmune components, as in the case of multiple sclerosis; genetic components, as in the case of leukodystrophies; and accidents related to risks at birth or traumas at any stage of life, including the aging process itself (Garde et al., 2000; Miller et al., 2012; Coggan et al., 2015; de Faria et al., 2021). Furthermore, several studies have correlated myelination deficits with neurodegenerative diseases and various mental illnesses (e.g., Prins and Scheltens, 2015; Koshiyama et al., 2020; Steadman et al., 2020; Wang et al., 2020), although the role of these deficits in the etiology of these conditions is still not entirely clear.

The phenomena of myelination and remyelination begin with the establishment of a dialogue and its maintenance between oligodendroglial lineage cells and axons. In general, the reciprocal recognition between an immature OL and the axon(s) that it will myelinate will culminate in their maturation, forming the wrapping myelin sheath. Both intrinsic and extrinsic signals initiate the myelination of a specific axon. Extrinsic signals, including neuronal activity and extracellular chemical signals, seem to be of great importance (Lee and Fields, 2009; Emery, 2010). Strategies aimed at strengthening the nervous system’s resistance to demyelination factors and promoting the myelination/remyelination process should include enhancing intercellular communication mechanisms that favor and/or increase the dialogue between OLs and neurons. A significant percentage of oligodendrocyte precursor cells (OPCs) remain homogeneously distributed throughout life in the adult brain. OPCs maintain their potential to differentiate into mature OLs and can proliferate and migrate to regions where they are required (Chang et al., 2000; Sherafat et al., 2021; Beiter et al., 2022). OPCs and axons interact through several mechanisms, including the establishment of both glutamatergic and GABAergic functional chemical synapses between axons and OPCs (Bergles et al., 2000; Lin and Bergles, 2004; Kukley et al., 2007; Káradóttir et al., 2008; Müller et al., 2009; Tanaka et al., 2009; Vélez-Fort et al., 2010; Orduz et al., 2015; Zonouzi et al., 2015). Moreover, there are regions where OPCs presynaptically contact neurons, affecting their functions (Zhang et al., 2021; Fang et al., 2022). The beginning of myelination bears important similarities with synaptogenesis, and it has been proposed that the establishment of these chemical synapses could correspond to potential myelination sites once the appropriate signals for maturation are present (Stys, 2011; Almeida and Lyons, 2014; Proctor et al., 2015). GABAergic communication seems to be directly correlated with the degree of maturation of OLs and the myelination process (Serrano-Regal et al., 2020). For example, in *in vitro* experiments the expression of GABA type A (GABA_A_) receptors in the OL membrane is controlled by their proximity to neurons (Arellano et al., 2016), whereas in *in vivo* experiments, potentiation of GABAergic pathways promotes myelination/remyelination of the system in a variety of experimental models and under different pharmacological conditions of modulation, including administration of tiagabine (Zonouzi et al., 2015), ganaxolone (Shaw et al., 2019), baclofen (Serrano-Regal et al., 2022) or n-butyl-β-carboline-3-carboxylate (β-CCB; Cisneros-Mejorado et al., 2020). This last example represents a case of great interest because it has been shown that in *in vitro* experiments β-CCB acts strongly by potentiating GABA_A_ receptor activity in OLs, with no significant effect on the receptor expressed in cortical neurons (Arellano et al., 2016; Cisneros-Mejorado et al., 2020; Ordaz et al., 2021), a condition that is essential in the development of drugs with potential clinical use for myelination. Systemic administration of β-CCB (Cisneros-Mejorado et al., 2020) could influence remyelination through indirect mechanisms independent of its action on oligodendroglial GABA_A_ receptors. Thus, given the importance of developing molecules with specific actions on GABAergic signaling in myelination, in this study we explore in greater detail the possibility that systemically administered β-carbolines act directly on GABA_A_ receptors expressed in oligodendroglial cells, which causes changes in the maturation dynamics to regenerate myelin sheaths. For this purpose, we analyzed in parallel the effect of three different β-carbolines on the ability to remyelinate the previously demyelinated caudal cerebellar peduncle by ethidium bromide (EB) stereotaxic injection (DRICP model), as it has been previously shown (Woodruff and Franklin, 1999; Cisneros-Mejorado et al., 2020). For each β-carbolines studied we know its effect on the endogenous receptor of OPCs and on receptors expressed in cortical neurons maintained *in vitro* (Ordaz et al., 2021), thus we can compare their relative potency on the receptor response and their possible effect on the myelination process. The β-carbolines used were: (1) β-CCB, a robust potentiator of the GABA_A_ receptor response in OLs that has no effect on cortical neurons; (2) β-CCE which has a moderate potentiation effect on the GABA_A_ receptor in OLs without affecting the neuronal receptor; and (3) DMCM which negatively modulates both receptors. Changes in myelination in the DRICP model were tracked using MRI and specific staining with Black-Gold II (BGII) in animals that received or not one of the three β-carbolines. In addition, the state of the myelin structure was directly evaluated by determining the axonal *g*-ratio using toluidine blue staining. Moreover, the OPC and mature OL populations were quantified under the different experimental conditions using immunodetection. We found that β-carbolines with positive allosteric modulation at oligodendroglial GABA_A_ receptors promoted effective remyelination in the DRICP model, through an increase in the number of OPCs and mature OLs in the injured area.

## 2 Materials and methods

### 2.1 Study approval

All experiments were performed according to the procedures approved by the Ethics Committee of the Instituto de Neurobiología, Universidad Nacional Autónoma de México. The *in vivo* experiments were conducted using male Sprague–Dawley (SD) rats (280–310 g, 10–12 weeks) in accordance with the Guide for the Care and Use of Laboratory Animals (National Institutes of Health). Animals were kept under conventional housing conditions (22 ± 2°C, 55 ± 10% humidity, 12 h day/night cycle and *ad libitum* access to water) at the Instituto de Neurobiología, Universidad Nacional Autónoma de México. All possible efforts were made to minimize animal suffering and the number of animals used, and the procedures complied with ARRIVE guidelines.

### 2.2 Demyelination and remyelination of inferior cerebellar peduncle (DRICP model)

Rats were anesthetized with ketamine/xylazine (a mixture of 70 mg/Kg and 6 mg/Kg, respectively, dissolved in saline solution) and then positioned on a stereotaxic instrument (Stoelting Co., Wood Dale, IL, United States) in a surgery room at room temperature. Demyelination was induced by injecting 2 μL of 0.05% ethidium bromide (EB) into the inferior cerebellar peduncle (*i.c.p.*) of male rats in accordance with the Paxinos atlas coordinates (Paxinos and Watson, 2007) as follows: AP -2.28 mm (from interaural), LR 3.2 mm (from midline), H 7.6 mm, and the incisor bar positioned 3.5 mm below the center of the aural bars. Control animals were injected with an equal volume of sterile saline solution.

### 2.3 Black gold II (BGII) staining

For histological examination, rats were transcardially perfused with 0.1 M phosphate buffered saline (PBS) (pH 7.4) followed by 4% paraformaldehyde (PFA) in the same buffer. Subsequently, the brains were dissected. For tissues that were processed for toluidine blue, the fixation buffer included 2% glutaraldehyde. Then, to cryopreserve them, they were placed in a PBS-30% sucrose solution (50 mL PBS/15 g sucrose) for 36–48 h. Coronal or parasagittal sections with a thickness of 40 μm were obtained in a cryostat (Leica CM 1850) at a temperature between −24°C to −26°C and suspended in PBS. Sections were incubated in BGII solution (0.3%) for 15–20 min at 65°C. Subsequently, they were washed with PBS for 2 min (twice), followed by 1% sodium thiosulfate (Na-Thio) for 3 min at 65°C and finally washed with PBS for 2 min (twice). Then, to perform the assembly, xylol was added for 1 min, and later DPX medium was added, and a coverslip placed. Tissue sections were visualized under a microscope, and representative images were acquired with a Leica ICC50 HD camera (Leica Microsystems, Wetzlar, Germany). Relative intensity of myelin BGII staining (RIS) in the *i.c.p.* was quantified using ImageJ software (version1.52i). After converting the images to grayscale, relative intensity was obtained from a given region of interest (ROI), calculated by normalizing intensity values from each ROI against the background intensity value from each section (Hakkarainen et al., 2016; Cisneros-Mejorado et al., 2020), applying the following relationship: RIS = (intensity of background − mean intensity of labeling in ROI) / intensity of background.

### 2.4 Toluidine blue staining

After fixation and brain dissection (see above), tissue blocks encompassing the *i.c.p.* were cut. Samples were postfixed by incubating for 2 h with 3% glutaraldehyde in 0.1 M cacodylate buffer (Electron Microscopy Sciences Co., EMbed-812), pH 7.4, at 4°C for 60 min, then washed with 0.1 M sodium cacodylate pH 7.4. for 30 min (twice), dehydrated in increasing concentrations of ethanol and then immersed in propylene oxide (2 × 30 min at room temperature) to the interface solvent. Samples were infiltrated for 24 h with plastic resin using a 1:1 dilution of complete Epoxy (Luft) resin with propylene oxide, then transferred to undiluted resin and mixed on a rotatory shaker for 24 h. Tissue blocks were transferred to flat silicone rubber molds (EMS) for orientation, filled with epoxy resin (Luft), and polymerized by incubating at 60°C for 36 h. Semithin sections (600 nm) were cut with a glass knife using an ultramicrotome (RMC Model MTX, Boeckeler Instruments Inc.) and dyed with 1% Toluidine Blue O diluted in sodium tetraborate at 90°C, then washed with distilled water and incubated in xylol for 1 min. Later, DPX medium was added, and a coverslip placed. Tissue sections were visualized under a light microscope, and representative images (10X, 40X or 100X) were acquired with a Leica ICC50 HD camera (Leica Microsystems, Wetzlar, Germany). Axon morphometry was analyzed using AxonSeg program to measure parameters of interest, like myelin thickness, and estimate the *g*-ratio defined as *g =* axon diameter / (axon diameter + myelin thickness). In this case, the quantified parameters were estimated on the periphery of the lesion, where axons were better resolved.

### 2.5 Magnetic resonance

Anesthesia was induced with isoflurane (4–5% in compressed air) using an anesthetic chamber and maintained under 2% with a facemask during the procedure. T2-weighted imaging and diffusion MRI (dMRI) protocols were performed at the National Laboratory for Magnetic Resonance Imaging with a 7 T magnet (Bruker Pharmascan 70/16US) using similar protocols to those reported previously (Cisneros-Mejorado et al., 2020). Briefly, data sets for dMRI were acquired using a spin-echo, single-shot echo-planar imaging sequence with the following parameters: slice thickness = 0.65 mm, no inter-slice gap, no physiological gating, TR = 2 s, TE = 26 ms, FOV = 25 × 25 mm, image size = 167 × 167 mm, resolution = 150 × 150 mm, 40 diffusion directions, b = 650 s/mm^2^, 1 average. T2-imaging was acquired with the following parameters: slice thickness = 0.5 mm, no inter-slice gap, TR = 2.5 s, TE = 33 ms, FOV = 30 × 20 mm^2^, image size = 250 × 170 mm^2^, resolution = 120 × 118 mm^2^, 9 averages and 28 slices. Pre-processing of dMRI data sets included both reduction of motion and eddy current-induced geometric distortions by linear transformation of each volume to the average non-diffusion weighted volume and denoising via random matrix theory (Veraart et al., 2016). Total scan time was 36 min per animal. The MRtrix software package^1^ was used to estimate the tensor model in the dMRI protocol, from which we derived fractional anisotropy (FA) apparent diffusion coefficient (ADC), principal diffusion vector (PDV), and radial and axial diffusivities (RD and AD, respectively). dMRI parameters were analyzed using *i.c.p.* ROI analysis was manually delineated on PDV images. In graphs, the laterality index was calculated for all metrics to show the dynamic of dMRI metrics, like: X’ = [(ipsilateral-contralateral)/(ipsilateral+contralateral)] × 100, where X’ is the corresponding map (FA, ADC, RD or AD).

### 2.6 β-carboline administration

For β-carboline *in vivo* administration, the vehicle as control or one particular β-carboline solution was prepared in aliquots and then coded alphanumerically by an independent researcher. First, either β-carboline β-CCB (1 mg/Kg) or β-CCE (1 mg/Kg) were dissolved in DMSO (in 0.5% of the final injection volume 200 μL) and subsequently in PBS (1X). DMCM (0.35 mg/Kg) was directly dissolved in PBS. The mixture was sonicated for 60 min at 37°C. Animals were randomly assigned to one treatment based on a blinded analysis design, and either vehicle alone or a β-carboline solution was administered intraperitoneally after 14 days of *i.c.p.* injection with either EB or saline solution. β-carboline administration was done every 24 h from day post-lesion (dpl) 14 until 28. Treatment with β-carbolines for the time and doses indicated did not cause obvious changes in animal behavior, such as freezing or signs of pain, anxiety, or aggressiveness, and did not show alterations under the exploratory behavioral test.

### 2.7 Immunohistochemistry

To analyze the OPC and OL populations, brain slices (40 μm) were immunoassayed with antibodies against oligodendroglial cell-specific markers: rabbit anti-NG2 (1:250; Millipore, AB5320) for OPCs and mouse anti-APC/CC1 (1:250; Sigma-Aldrich, OP80) for mature OLs. In all cases, fixed slices were washed and permeabilized in PBS with 0.1% Tween-20. Then, the slices were incubated in blocking buffer solution (PBS, 5% BSA, and 0.3% Triton X-100) for 30 min at 4°C. Incubation with primary antibodies (diluted in PBS containing 5% goat serum and 0.1% Tween-20) was performed overnight at 4°C. Slices were rinsed in PBS with 0.1% Tween-20 and incubated for 4 h at 4°C with Alexa fluorophore-conjugated: 1:300 goat anti-mouse IgG (H + L) conjugated with Alexa Fluor 488 (Invitrogen, A-11001) or 1:300 goat anti-rabbit IgG (H + L) conjugated with Alexa Fluor 594 (Abcam, ab150080) according to the host species of the primary antibodies. After five washes in PBS with 0.1% Tween-20, samples were stained with 4′,6-diamidino-2-phenylindole dihydrochloride (4 μg/mL DAPI, from Molecular Probes, D1306) to identify cell nuclei. In all cases, the absence of nonspecific interactions of secondary antibodies was corroborated by omitting the primary antibodies. Finally, the samples were mounted on Mowiol (Sigma-Aldrich, 81,381) and the preparations were visualized (20X) under an LSM880 laser scanning confocal microscope (Zeiss, Oberkochen, DE). For quantitative analysis, z-stack images were processed in ImageJ analysis software.

### 2.8 Substances

N-butyl-β-carboline-3-carboxylate (β-CCB), ethyl 9H-pyrido[3,4-b]indole-3-carboxylate (β-CCE) and 4-ethyl-6,7-dimethoxy-9H-pyrido[3,4-b] indole-3-carboxylic acid methyl ester (DMCM); were all obtained from Tocris Bioscience (Bristol, United Kingdom), isoflurane from PiSa Lab (PiSa, Guadalajara, JAL, México), and Permount mounting medium from Fisher Scientific (FS, Fair Lawn, NJ, United States). Salts, PFA, EB, xylenes and DMSO were acquired from Sigma-Aldrich Co. Glutaraldehyde, cacodylate buffer and osmium tetroxide was from Electron Microscopy Sciences, Hatfield, PA, United States (EMS).

### 2.9 Statistical analysis

All data are expressed as mean ± S.D. The means of two groups were compared using a Student’s t-test or, when appropriate, an analysis of variance with Fisher’s *post hoc* test were performed to compare between groups. To verify the normality of the data, the Kolmogorov–Smirnov test was employed, while the Levene test was used to test the homoscedasticity. Statistical analysis was performed using GraphPad Prism or Excel Office software. Differences were considered to be significant at *p* < 0.05.

## 3 Results

### 3.1 Demyelination of the inferior cerebellar peduncle (*i.c.p.*) by MRI and histology with BGII or toluidine blue semithin sections

**Figure 1:**
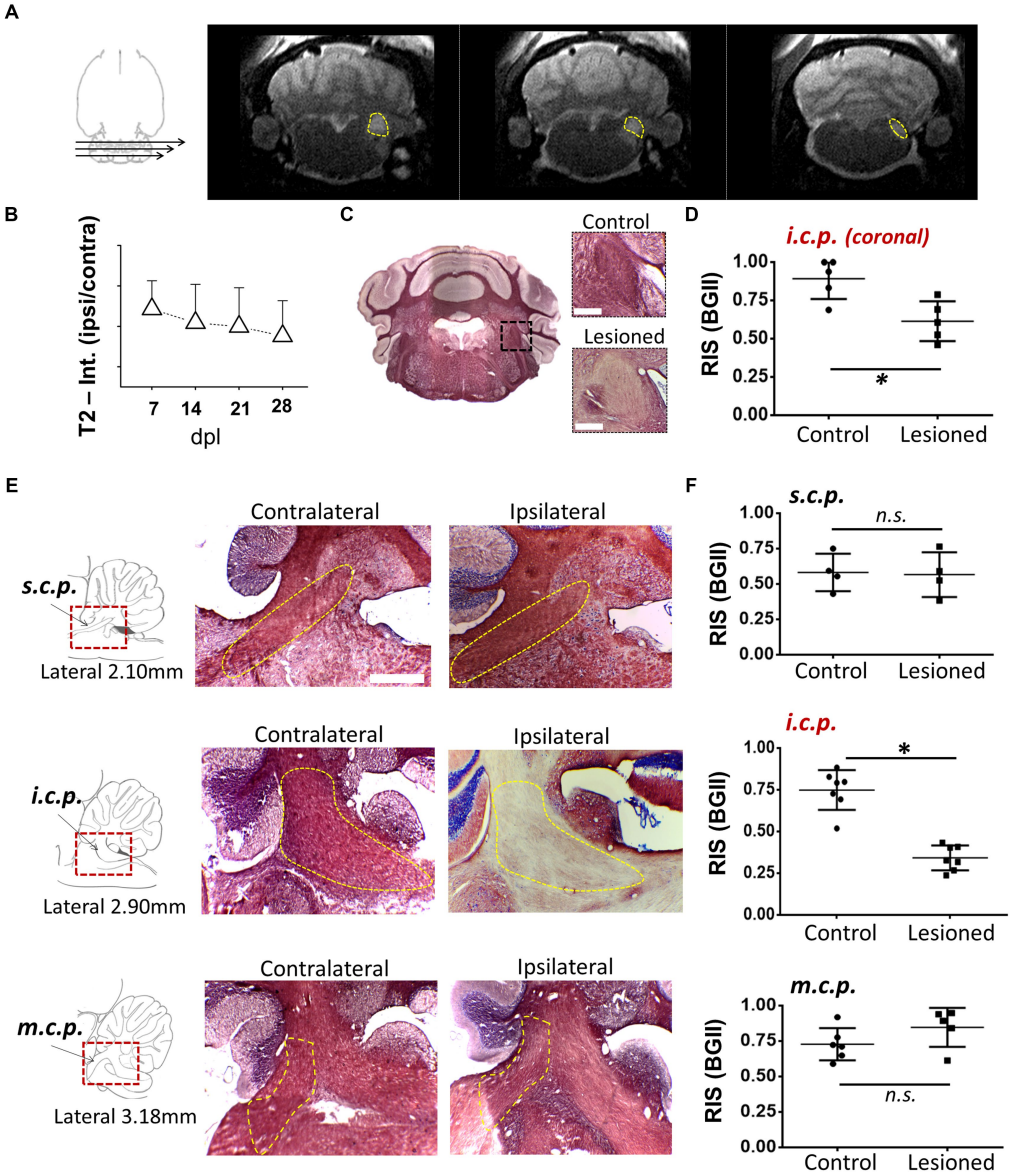
Demyelination lesion followed by BGII and MRI in the inferior cerebellar peduncle. **(A)** Representative T2-weighted images of an animal injected with EB (0.05%) in the inferior cerebellar peduncle (*i.c.p.*); the yellow dotted line indicates the *i.c.p.* region hyperintensity. **(B)** T2 signal quantification in the *i.c.p.* (ipsilateral/contralateral) from 0 to 28 dpl (*n* = 4–5 for each point). **(C)** Representative images of sections in the panoramic coronal plane stained with BGII of the cerebellum. Amplifications for both control (top insert) and lesioned (bottom insert) *i.c.p.* are also shown. Bar = 200 μM. **(D)** Relative intensity of staining (RIS) quantification in *i.c.p.* sections as illustrated in C (*n* = 4 for each case). **(E)** Images in the parasagittal plane stained with BGII including superior (*s.c.p.*), inferior (*i.c.p.*) and medial (*m.c.p.*) cerebellar peduncles from both contralateral and ipsilateral side to EB injection, as indicated. **(F)** RIS quantification for each peduncle. The RIS decrease in the lesioned *i.c.p.* was evident, while the RIS for *m.c.p.* or *s.c.p.* remained unaffected (*n* = 4 for each case). *p < 0.05. Data are the mean ± S.D., *t*-test was used.

As an experimental model to explore the mechanisms of remyelination and the possible effects of drugs that enhance it, we used the model of demyelination by EB injection in the *i.c.p.* of rats (Woodruff and Franklin, 1999; Cisneros-Mejorado et al., 2020). We first used MRI T2-weighted imaging (T2w) with 5 lesioned rats, to track the effectiveness of the demyelinating lesion caused by EB injection starting on day 7, this is a method that provided images consistent with the degree of peduncle demyelination, visualized as hyperintense zones that coincided with the *i.c.p.* area (Figures 1A,B, *N* = 4–5 for each point) and that remained affected for more than 28 dpl. BGII histology in coronal sections from lesioned rats (*n* = 4) revealed a statistically significant decrease in staining of more than 30% (34.1 ± 8.1%) compared to the contralateral side (control), consistent with a pattern of demyelination at the ipsilateral (lesioned) *i.c.p.* area (Figures 1C,D). The T2w analysis indicated an accurate regionalization of the lesion (Figure 1A), this was confirmed by BGII staining analysis in parasagittal sections of the *i.c.p.* and the superior (*s.c.p.*) and middle (*m.c.p.*) peduncles (*n* = 4), as shown in Figures 1E,F. The results indicated that the lesion caused by the stereotaxic injection of EB is mainly restricted to the area of the inferior peduncle (45.9 ± 7.4% decrease compared with the contralateral side), without effects on the *s.c.p.* or *m.c.p.*, as suggested by BGII staining quantification for each region at 28 dpl (Figure 1F).

**Figure 2:**
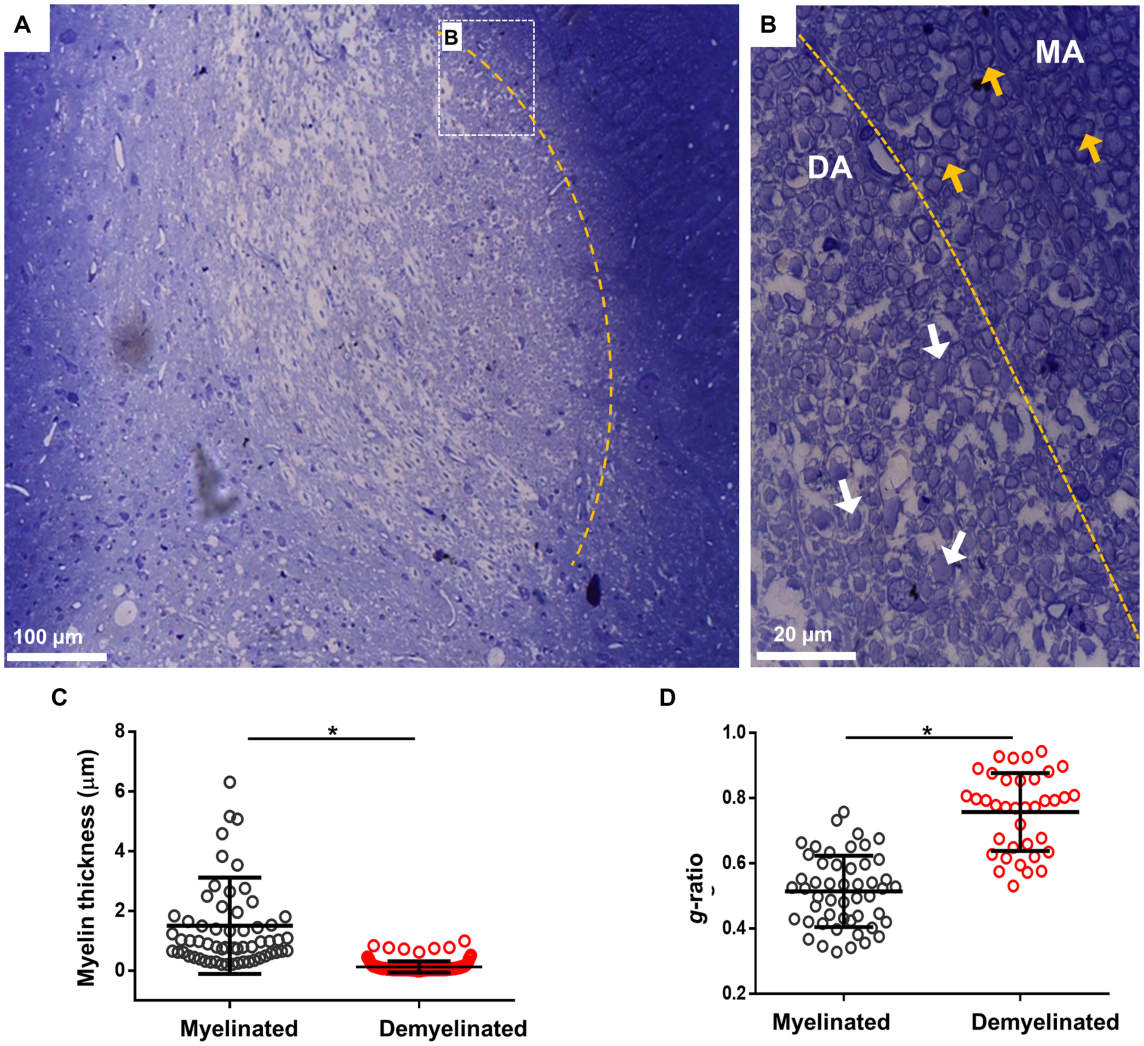
Toluidine blue staining of the *i.c.p.* lesioned by EB injection *in vivo*. **(A)** Image illustrates lesioned *i.c.p.* stained with toluidine blue, the yellow line signals the boundary between a low stained region that coincided with the lesioned *i.c.p.* area (center of image), while the adjacent area showed a strong staining. Rectangle B indicate boundary areas corresponding to amplified image to the right. **(B)** Amplification to illustrate that the lesioned *i.c.p.* exhibited a great number of demyelinated axons (DA, white arrows) compare with the adjacent area which mainly contained myelinated axons (MA, yellow arrows). **(C,D)** shows the myelin thickness and *g*-ratio quantification in MA and DA zones. More than 50 axons were quantified from sections obtained from different animals (*n* = 4 animals). Each group represents the mean (± SD) of the counted axons. ^*^p < 0.05, *t*-test was used.

Another technique used to analyze myelin structure was staining with toluidine blue, which allows the quantification of *g*-ratio changes. For this, *i.c.p.* sections of the EB-injected animals (*n* = 4) were processed at 28 dpl for toluidine blue and visualized in semi-thin 600 nm sections. As illustrated in Figure 2A, the lesion induced by EB injection caused a clear delimitation between the lesioned *i.c.p.* and the adjoining tracts, in this case the spinal trigeminal nerve. Amplification of representative areas are shown in Figure 2B. In the *i.c.p.* area, numerous axons were devoid of myelin (DA region, white arrows), while the intact region contained normal myelinated axons (MA region, yellow arrows). As this is shown in Figures 2C,D, myelin thickness decreased and *g*-ratio increased in the lesioned area, or compared with the control region (from 1.51 ± 1.06 μm to 0.22 ± 0.18 μm for myelin thickness, and 0.52 ± 0.04 to 0.75 ± 0.11 for *g*-ratio), indicating a significant structural.

### 3.2 Effect of β-carbolines on *i.c.p.* monitored with MRI

**Figure 3:**
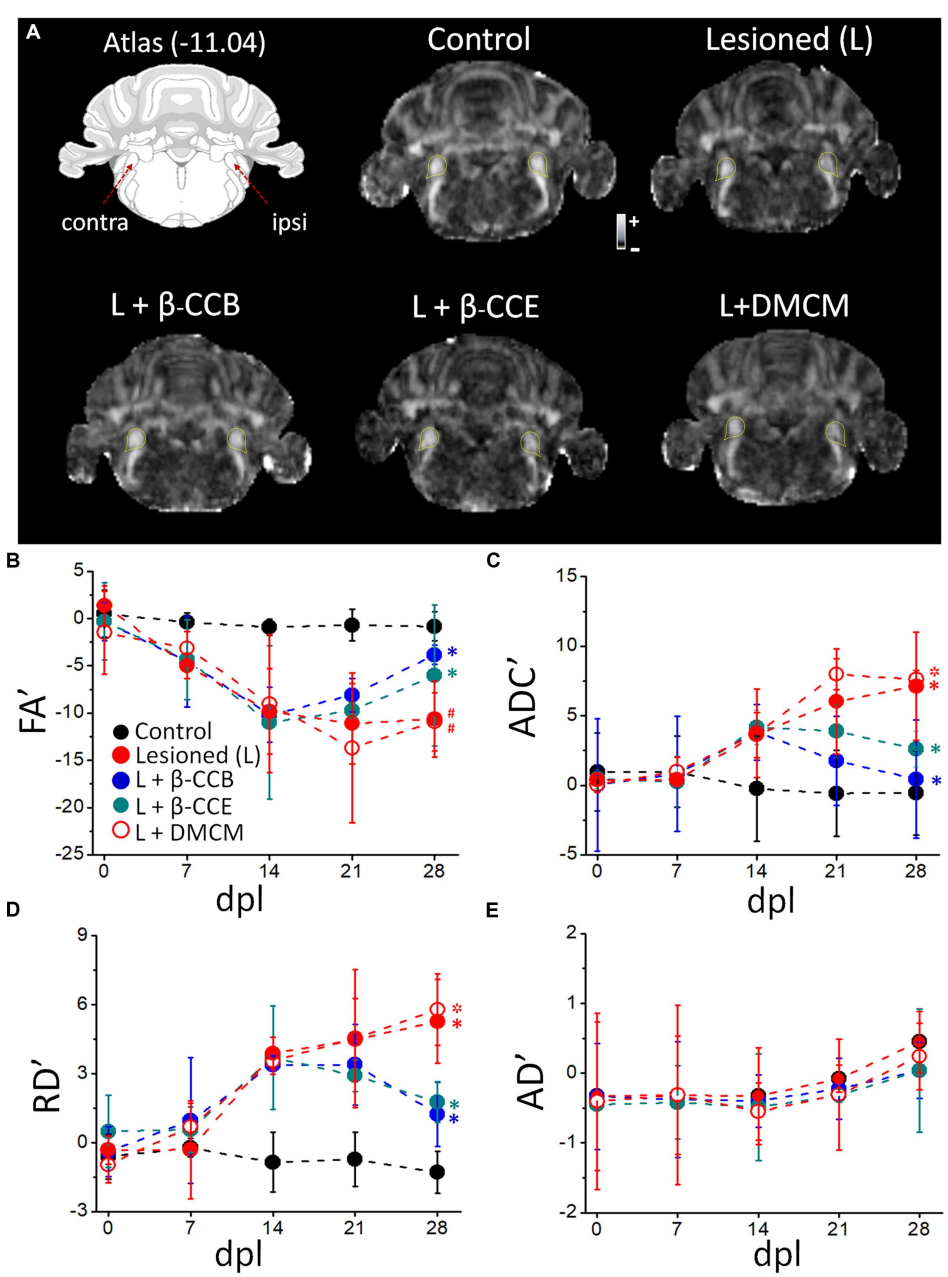
Longitudinal analysis of DTI metrics evaluated over the DRICP model time-course and the effect of β-carboline administration. **(A)** Images are representative FA maps acquired at 28 dpl in regions that include the *i.c.p.* as indicated in the diagram and delineated by the yellow dotted line in the images. Five experimental groups are illustrated: control group, lesioned (i.e., animals injected with EB in the ipsilateral *i.c.p.*) without treatment, and the lesioned group that was treated (L +) with β-CCB (1 mg/Kg), β-CCE (1 mg/Kg) or DMCM (0.35 mg/Kg). **(B)** The graphs represent the values of the laterality index for FA’ **(B)**, ADC’ **(C)**, RD´ **(D)** and AD´ **(E)** for the images captured on the indicated days of the experiment. Each data point represents the mean ± SD (*n* = 4 for each point. (ANOVA)) of the respective group (* or ^#^ indicates differences of the values at 28 dpl vs. 14 dpl or 28 dpl vs. 7 dpl, respectively, within the same group identified with the corresponding color, ^*^p < 0.05, ANOVA).

Once the time-course of myelin structural changes caused by EB injection was characterized, groups of animals were lesioned in similar conditions and analyzed longitudinally using dMRI protocols (Figure 3). This technique provided with a quantitative analysis of tissue microstructure by estimating maps for FA, ADC, RD and AD. Together, these parameters reflect the amount of myelin in the tract and infer axon integrity, providing data about the status of the white matter in both normal and pathological conditions (e.g., Song et al., 2002, 2003, 2005; Concha et al., 2006; Kumar et al., 2009; Laitinen et al., 2010, 2015). MRI diffusion protocols were applied every 7 days from day zero, when the lesion was made, until day 28 dpl. Figure 3A depicts the diagram of the studied region (Paxinos and Watson, 2007) and representative images of the FA of experimental groups analyzed at 28 dpl, as follows: (1) control (non-lesioned, *n* = 4), (2) lesioned without treatment (*n* = 4) and, (3) lesioned and treated with a β-carboline, either β-CCB, β-CCE or DMCM (*n* = 4 for each case). The yellow lines in each image correspond to the region indicated to the *i.c.p.* in the corresponding atlas.

First, we analyzed the time-course of the changes observed throughout the experiment, acquiring MRI images for analysis from day zero, before EB injection, and every week until 28 dpl (Figure 3). As shown, control values (black circles) for both FA and ADC remained stable from day 0 to 28 dpl, while in the lesioned group (red circles) FA began to decrease at 7 dpl and continued until 14 dpl. FA remained stable in this group from 14 dpl to 28 dpl (Figure 3B). On the other hand, ADC showed an increase at 14 dpl and then from 21 dpl to 28 dpl (Figure 3C). Similarly, RD showed an increase from 14 dpl to 28 dpl (Figure 3D). In contrast, AD showed no significant differences between the lesioned and control *i.p.c.* within the same time frame (Figure 3E).

**Figure 4:**
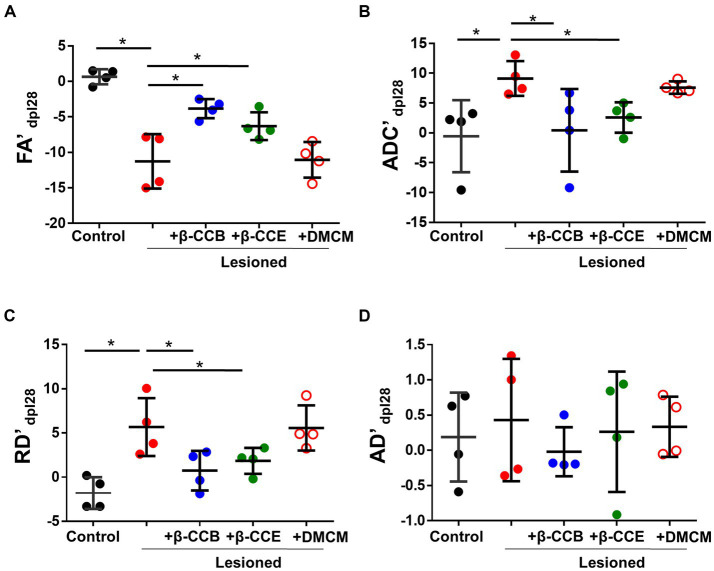
DTI metrics of the DRICP model and the effect of β-carboline administration. Graphs show quantification of the FA’ **(A)**, ADC’ **(B)**, RD´ **(C)** and AD´ **(D)** metrics expressed as the laterality index calculated for each subject and every parameter at 28 dpl as defined in the methods section. Data represent the mean ± SD (*n* = 4) for each experimental group as illustrated in Figure 3 (^*^p < 0.05, ANOVA).

Then, we compared each metric (laterality index, as indicated in methods section) between groups at the end of the experiment (28 dpl). As shown in Figure 4, EB injection induced a more than 10-fold decrease in the FA index (from −0.82 ± 1.54 to −10.63 ± 1.6) compared with the control and a more than 8-fold and 4-fold increase in the ADC (from −0.55 ± 2.33 to 9.12 ± 2.34) and RD (from −1.28 ± 1.04 to 5.27 ± 1.28), respectively, compared with the control. This result strongly suggests an alteration of white matter that was compatible with myelin loss. Also, the AD map did not change significantly, suggesting that there was no axonal degeneration. However, lesioned animals that were treated with β-carbolines showed differential effects depending on the substance they received. Both β-CCB and β-CCE groups showed a statistically significant recovery of all calculated metrics, as the FA index increased, and the ADC and RD decreased. The DMCM group did not show any effect and its metrics like those displayed by the demyelinated group.

Parallel monitoring and MRI analysis was carried out in lesioned animals that were treated from 14 dpl to 28 dpl with either β-CCB (blue circles), β-CCE (green circles) or DMCM (empty red circles). With respect to FA and ADC values and from 14 dpl to 28 dpl, the β-carboline groups showed differential changes when compared with the lesioned group that was not treated, as shown in Figures 4A,B. Both groups treated with β-CCB or β-CCE showed an FA increase and an ADC decline. For both metrics, the most robust changes were observed for β-CCB treatment, while the β-CCE treatment comparatively generated an attenuated effect (Figures 4A,B). Thus, β-CCB treatment analyzed at 28 dpl showed an FA value statistically different from that of the lesioned group without treatment and showed no difference when compared to the non-lesioned control group, a result that was similar for ADC analysis. On the other hand, treatment with β-CCE caused an FA increase and an ADC decrease, but in this case only the FA metric was statistically different compared to the control lesioned group. In contrast, systemic DMCM treatment did not show differences in FA at 28 dpl compared to the control lesioned animals but showed a greater ADC increase to that observed in the control group. In addition, diffusivity parameters calculated in the analysis were consistent with these results; thus, the RD values for the DMCM group was similar to those of the lesioned group without treatment, maintaining a robust increase with respect to the non-lesioned group at 28 dpl, while both β-CCB and β-CCE showed a decrease in RD that seemed to start at 21 dpl and continued at 28 dpl when this metric was statistically different from the lesioned control group (Figure 4C). In all cases, treatments with β-carbolines did not show significant changes in AD metric (Figure 4D).

### 3.3 β-carboline effect on the DRICP model analyzed with BGII

**Figure 5:**
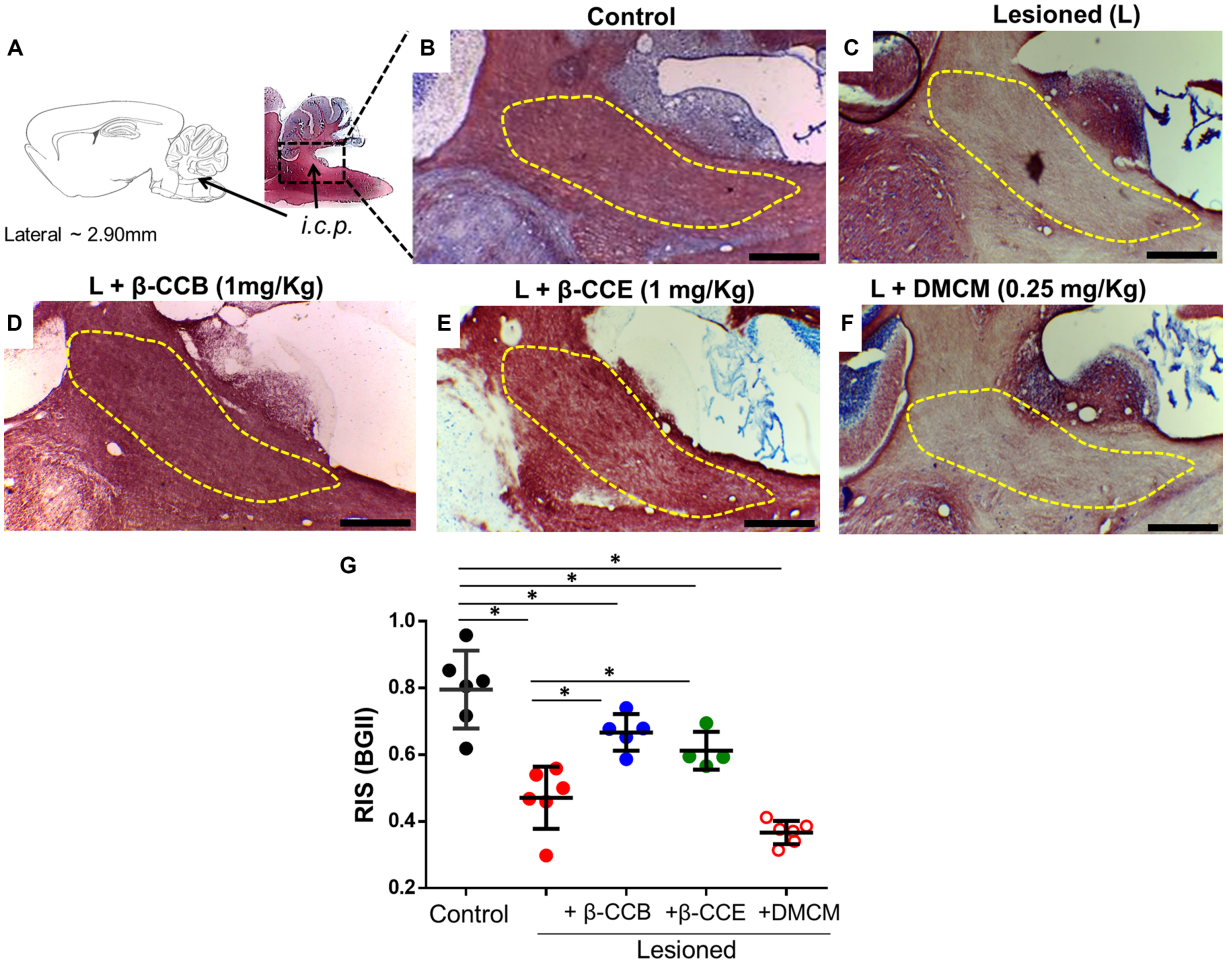
DRICP model evaluated using BGII staining and analysis of the effect of β-carboline administration. **(A)** Diagram of the *i.c.p* area analyzed by BGII staining at 28 dpl. B-F. Representative amplified images of the *i.c.p.* region for the distinct experimental conditions indicated in Figure 3: control **(B)**, lesioned **(C)**, and lesioned group that was treated with β-CCB [1 mg/Kg, **(D)**], β-CCE [1 mg/Kg, **(E)**], or DMCM [0.35 mg/Kg, **(F)**]. **(G)** Graph shows the *i.c.p.* relative intensity stain (RIS) for each experimental group at 28 dpl (*n* = 4 for each case). ^*^p < 0.05 vs. control group (ANOVA).

Once the complete dMRI analysis was complete at 28 dpl, all brains were processed in parallel for histology with BGII staining. Representative images of samples cut in the parasagittal plane for each group are illustrated in Figures 5A–F. The diagram in Figure 5A illustrates the analyzed *i.c.p.* area. In Figure 5B, this area (yellow line) is shown stained under control conditions (*n* = 4) using the BGII technique. Figures 5C–F depict sections from animals lesioned under the DRICP model either without β-carboline treatment (*n* = 4, Figure 5C) or treated with β-CCB, β-CCE, or DMCM (*n* = 4 for each case, Figures 5D–F). A significant decrease in BGII staining was detected in the *i.c.p.* area of lesioned animals (compare Figure 5B vs. Figure 5C). However, the lesioned *i.c.p.* from animals that were treated with β-CCB or β-CCE revealed an increase in BGII staining, while DMCM administration did not produce any improvement. This was determined by calculating the RIS parameter (see methods) that was plotted in Figure 5G for each group. The estimated RIS indicated that both the untreated lesioned group and the DMCM-treated group showed a significant decrease in RIS compared to the control group, while the groups treated with either β-CCB or β-CCE showed a significant improvement. RIS values presented an increase that was statistically different to that observed in the lesioned group without treatment, although the values at 28 dpl remained different compared to the control group.

### 3.4 Myelin thickness analyses of lesioned *i.c.p.* treated with β-carbolines

**Figure 6:**
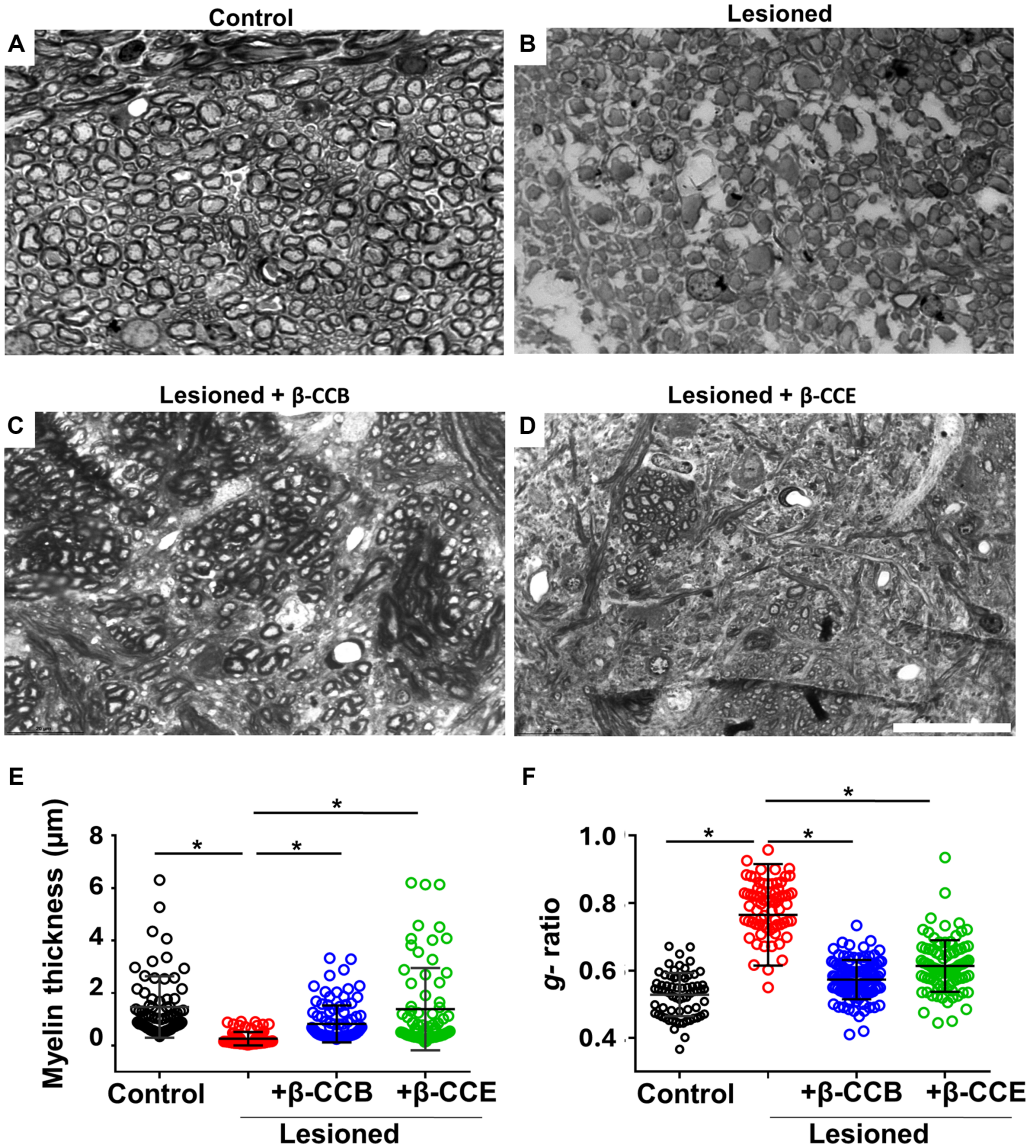
Axonal effect and myelin morphometric analysis at 28 dpl and β-carboline effect on the DRICP model. **(A–D)** Images illustrate *i.c.p.* semithin sections (600 nm thickness) stained with toluidine blue in control animals **(A)**, lesioned animals **(B)**, and lesioned animals treated with 1 mg/Kg β-CCB **(C)** or β-CCE **(D)**. **(E,F)** Show the myelin thickness and *g*-ratio quantification in all the experimental groups analyzed. More than 100 axons were quantified from sections obtained from different animals for each group. Bar = 20 μm. ^*^p < 0.05(*n* = 3–4; ANOVA).

Altogether, the results described above indicated that *i.c.p.* lesioned animals that were systemically treated with either β-CCB or β-CCE showed a clear improvement in parameters that correlated with robust *i.c.p.* remyelination. To obtain detailed information related to myelin structure, we processed brain tissue sections from the groups treated with β-carbolines for toluidine blue staining. For the analysis, we used AxonSeg software to estimate myelin diameter and *g*-ratio in each case. Figure 6 illustrates representative sections for each one of the four groups, as follows: control (*n* = 4, non-lesioned), lesioned group (*n* = 4), and lesioned group treated with β-carbolines, either β-CCB (*n* = 4) or β-CCE (*n* = 3). In the control *i.c.p.* region, there was a large population of myelinated axons (Figure 6A) that presented a mean myelin thickness of 1.47 ± 1.01 μm and a *g*-ratio of 0.52 ± 0.06 (Figures 6E,F). However, in brain sections from the lesioned group (Figure 6B) the myelin thickness decreased by more than 80% to 0.26 ± 0.15 and the *g*-ratio increased to 0.76 ± 0.1, an almost 1.5-fold increase compared with control group. In agreement with the results presented in previous sections, treatment with β-CCB and β-CCE promoted a significant recovery of myelin thickness to 0.98 ± 0.5 μm and 1.28 ± 1.06 μm, respectively, as well as a decrease in the *g*-ratio to 0.55 ± 0.05 and 0.58 ± 0.07, respectively. This result strongly suggested an improvement of the myelin structure compared with the untreated lesioned group.

### 3.5 Effect of β-carboline treatment on OPCs and mature OLs population within the lesioned *i.c.p.*

**Figure 7:**
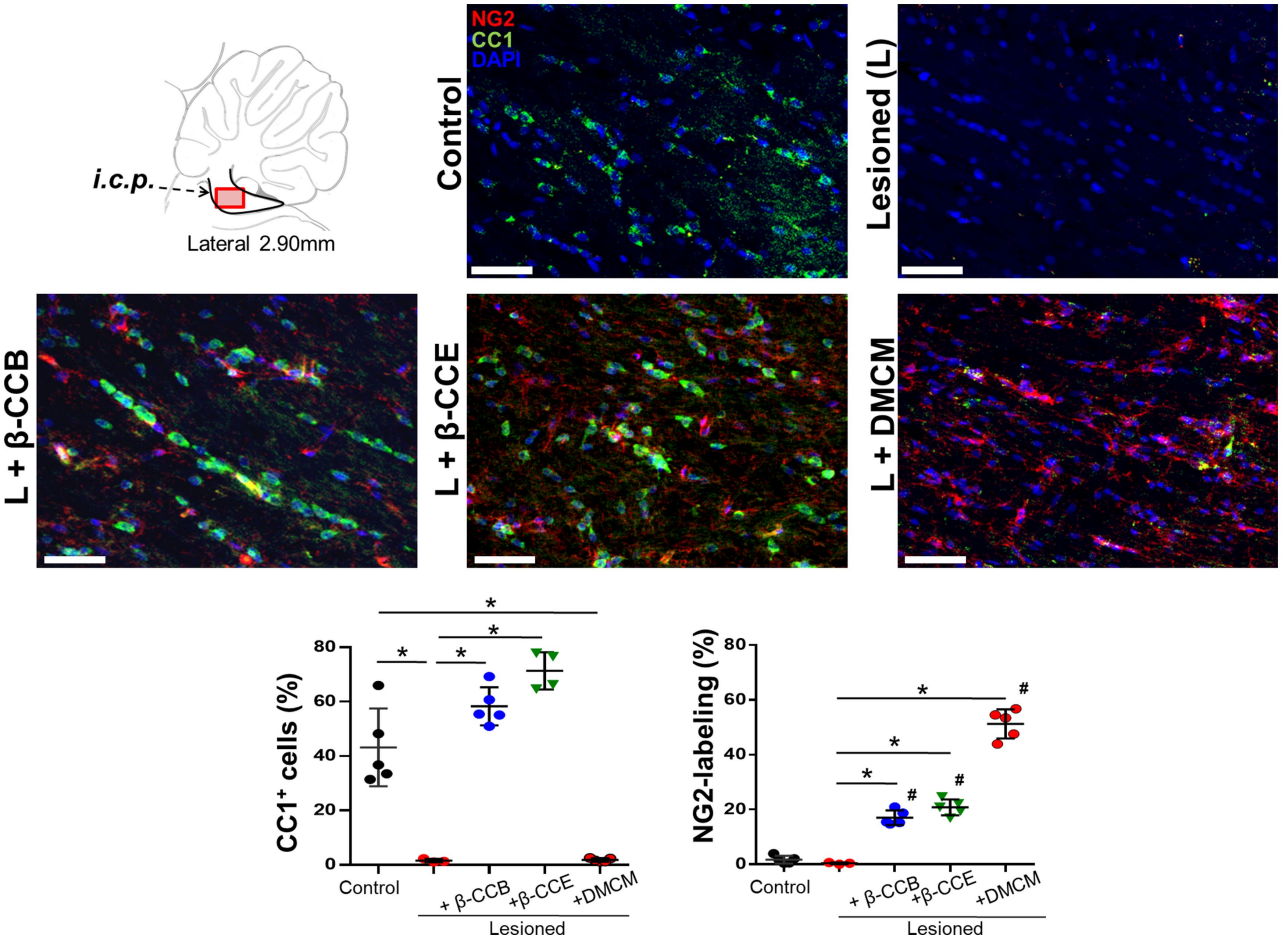
Status of CC1^+^ and NG2^+^ cells population in the *i.c.p.* at 28 dpl under the DRICP model and the effect of β-carbolines treatment. Diagram that indicates sagittal sections of the *i.c.p.* region that were immunolabeled and visualized using confocal microscopy. Antibodies against CC1 (specific for mature oligodendrocytes, in green) and NG2 (to label OPCs, in red) were co-labeled with DAPI (in blue). Representative images correspond to sections from the distinct experimental groups as indicated (bar = 400 μm). Graphs show quantification of the percentage of CC1^+^ cells or NG2^+^ cells labeled in each of the experimental groups analyzed: control, lesioned, and lesioned that was treated with one of the β-carbolines studied, as indicated. Columns represent the mean ± SD obtained from the analysis of sections from 3 subjects in each group (^*^p < 0.05).

Our results seem to indicate that β-carboline treatment enhanced the quantity of mature OLs in the lesioned area, which would be consistent with an increase in myelin content, this might be due to a concomitant increase in OPCs population and promotion of their maturation. To investigated whether this might be the case, in brain sections of the different groups (at 28 dpl), we studied both OPCs and mature OLs populations followed by double immunolabeling against NG2 and CC1, respectively (together with DAPI to identify nuclei). Figure 7 shows representative images for this analysis. First, the control *i.c.p.* revealed a robust CC1^+^ signal (in green) with low labeling for NG2^+^ (in red), this pattern changed in the lesioned group, where there was a strong decrease in the CC1^+^ signal and only a marginal change in NG2^+^. These effects were quantified by estimating the proportion of labeled cells with the respective immunomarker and considering the number of nuclei as the total number of cells (Figure 7). Thus, the CC1^+^ cells proportion decreased from 43.1 ± 14.3% to 3.6 ± 1.7% in the lesioned *i.c.p.*, while the NG2^+^ cells proportion presented no change. Different results were obtained from the lesioned group treated with β-carbolines. For example, administration of the GABA_A_ receptor-positive modulators, either β-CCB or β-CCE, produced a mean increase of the CC1^+^ signal (58.2 ± 7% and 70.3 ± 7.1%, respectively) in both cases, enhancing the proportion of CC1^+^ cells close to control values. DMCM administration, on the other hand, did not increase the proportion of CC1^+^ cells compared with the lesioned group, and the proportion of CC1^+^ cells remained low (i.e., 1.81 ± 2.2%).To determine whether β-CCB and β-CCE generated an increase in the proportion of CC1^+^ cells through a mechanism that could involve the maturation of precursor cells, we quantified the proportion of NG2^+^ cells in the same preparations. As mentioned above, the OPCs population remained in similarly low values in control and lesioned (without treatment) groups with 1.7 ± 1.4% and 0.4 ± 0.3%, respectively (Figure 7). Remarkably, both β-CCB and β-CCE promoted a significant increase in the proportion of NG2^+^ cells to 17 ± 2.7% and 20.75 ± 2.9%, respectively. Unexpectedly, DMCM administration in the lesioned group was even more effective in enhancing the NG2^+^ cell population (to 51.02 ± 5.3%). Together, these results suggested that, at 28 dpl, a spontaneous remyelination in the lesioned *i.c.p.* was affected by low OPCs density. Furthermore, the results supported the idea that although all three β-carbolines promoted an increase in the number of OPCs in the lesioned area, only β-CCB and β-CCE induced OPCs differentiation towards mature OLs.

## 4 Discussion

The results described here show that three distinct β-carbolines have differential effects on the time-course of cerebellar peduncle remyelination after being demyelinated by stereotaxic injection of EB (DRICP model). The process of myelin recovery after injury was followed both longitudinally by MRI analysis and transversally by BGII histology at the end of the experiment. These two methodologies support the idea that two of the β-carbolines studied, β-CCB and β-CCE, promoted the recovery of myelin content in the affected area, while DMCM had no effect on the myelin content assessed using these techniques.

OPCs survival in the CNS throughout lifespan, represents an opportunity to try to use their differentiation potential against pathologies that produce a detriment of myelin content in the system, this as a consequence of a decrease in the survival of mature OLs and/or a decrease in their turnover rate in essential development stages or in various physiological or pathological sceneries. Thus, controlling the myelination process by increasing the differentiation rate of OPCs towards mature OLs is a reasonable strategy against demyelinating diseases. However, most of the existing therapies, although with reasonable effectiveness, in most cases fail to completely correct the decline of the system, so further research is required in the search for new substances and/or strategies that might represent more effective treatments. In this context, we have argued that studying the communication mechanisms between neurons and OPCs is essential for recognizing the molecular events that lead to the dialogue that these two cell types will establish and the eventual differentiation of OPCs into mature OLs. The participation of the GABAergic system as a modulator in this dialogue has been supported by various studies carried out in different models, from *in vitro* preparations to whole animal models. Studies carried out in *in toto* models show that increasing GABAergic tone (Zonouzi et al., 2015) or the administration of a GABA_A_ receptor potentiator, such as a neurosteroid (Ghoumari et al., 2003; Shaw et al., 2019; Mouihate and Kalakh, 2020), have a positive effect on the degree of myelination in response to events that limit or even eliminate it. However, pharmacological manipulations in these studies use non-specific tools of the GABAergic system, which equally affect the receptors expressed in neurons and those expressed in OLs and other cells of the system, this makes it challenging to analyze the effects observed, and this is frequently complicated due to undesirable effects of the drugs used. Thus, achieving specific stimulation of oligodendroglial GABA_A_ receptors is a goal that could provide a tool that helps to understand the role of GABA as a modulator of the myelination process and the mechanisms that are involved in it, and obviously would direct to potential therapeutic drugs.

Recently, we have shown that in rodents, the main GABA_A_ receptor expressed in OPCs and OLs, isolated in the perinatal stage and maintained in culture, presents distinctive characteristics to the main subtypes expressed in neurons, especially these receptors do not contain the γ2 subunit, but mainly the subunit γ1, and its characteristics indicate the main presence of α3 and/or α2 subunit, combinations that are congruent with transcriptomic analyses of oligodendroglial cells (Larson et al., 2016; Ordaz et al., 2021). The GABA_A_ receptor of OLs presents differential effects to various substances such as β-carbolines compared to those observed on GABA_A_ receptors of cortical neurons maintained in culture (Arellano et al., 2016; Cisneros-Mejorado et al., 2020; Ordaz et al., 2021). In particular, β-carbolines originally have been described as inverse agonists on neuronal receptors acting through the benzodiazepine binding site (Peña et al., 1986; Rigo et al., 1994), however some of them such as β-CCB, cause a robust potentiation of the response to GABA in the receptor expressed in OLs, while in cortical neurons this drug generally has no effect or slightly decreases the response.

Taking advantage of the differential effects of β-carbolines on neuronal and oligodendroglial GABA_A_ receptors could provide a tool to specifically stimulate or inhibit pathways. With this purpose, we previously analyzed the effect of β-CCB on the remyelination process in the DRICP model. We observed that systemic β-CCB administration promotes remyelination of the damaged area. However, given that β-CCB administration was systemic, remained many questions about the drug action mechanisms, for example, especially if its promyelinating effect was related to the potentiation that it exerts on the oligodendroglial GABA response, and importantly, about the mechanisms that the activation of the receptor exerts to promote myelination. Here we systematically addressed the first question by administering two β-carbolines with different potencies on the receptor, as demonstrated in *in vitro* studies, to compare them with β-CCB in terms of their ability to promote remyelination in the DRICP model. We complemented the analysis using MRI and BGII techniques and estimating myelin thickness and the *g*-ratio under the different experimental conditions. Also, to obtain information for the second question, we analyzed the status of the oligodendroglial lineage in the myelin recovery stage, quantifying the population of OPCs (NG2^+^ cells) and mature OLs (CC1^+^ cells) by immunohistochemistry.

First, the promyelinating effect of β-CCB administration was confirmed in experiments in which injured animals of the DRICP model showed a significant improvement in parameters suggestive of remyelination obtained by MRI and BGII staining. β-CCB is among the β-carbolines that causes the greatest potentiation in the response to GABA expressed in OPCs and OLs *in vitro* (Ordaz et al., 2021) and has no significant effect on the GABA response in cortical neurons. We then tested the effects of β-CCE, which has a less potent effect on the receptor (about 50% of the effect of β-CCB at a concentration of 10 μM), and DMCM which causes inhibition of both neuronal and oligodendroglial responses. Both the analysis of myelin content studied by MRI and BGII is consistent with the idea that while β-CCE promoted myelin recovery, and DMCM administration had no effect on recovery of the lesioned area. This agrees with that expected considering the direction of the modulation of these drugs on the oligodendroglial GABA_A_ receptor, while β-CCB and β-CCE potentiate the GABA response, DMCM acts as a classic inverse agonist inhibiting the response in the oligodendroglial GABA_A_ receptor. It is also consistent with the fact that β-CCB appears to be more potent in its promyelinating action, however, in many aspects the effect of β-CCE is similar to that of β-CCB. In general, the recovery of the FA, ADC and RD parameters in the dMRI analysis indicates that, at 28 dpl, the animals treated for 2 weeks with β-CCB or β-CCE reached values that were close to the control before EB injection, while in the DMCM-treated group this recovery was not observed.

These data are consistent with the analysis performed with the BGII technique, given that the MRI analysis suggested a myelin content recovery. An increase in BGII staining was also indicative of myelin recovery in the animals treated with β-CCB or β-CCE; DMCM had no effect. Together the results strongly suggested that the ability of β-carbolines to promote remyelination is associated with their positive modulatory effect on the oligodendroglial GABA_A_ receptor. To reinforce the idea that the analyzed parameters are directly reflected on the myelin structure, we carried out a morphometric study of myelin thickness and the *g*-ratio in the lesioned region for the different groups treated with β-carbolines at 28 dpl. Again, these data showed that the typical parameters measured for myelin, such as thickness and *g*-ratio, were recovered with β-CCB and β-CCE treatment. It is important to note that both parameters were quantified in regions with well-resolved axons, this was extensive in the case of treatments with β-CCB, while with β-CCE there were regions that still remained with a scare presence of myelinated axons, observation that again suggested a direct correlation between the promyelinating effect potency and the corresponding potentiation of the β-carboline administrated.

Finally, the immunohistochemical analysis of OPCs and mature OLs populations in the injured area provided insight into the possible mechanisms involved in the promyelinating effect of β-CCB and β-CCE. As shown, both β-carbolines induced an increase in the number of NG2^+^ cells and CC1^+^ cells, all together, this suggested that these β-carbolines acting as positive modulators of the main GABA_A_ receptor expressed in OPCs increased their proliferation and differentiation to mature OLs. However, it is very important to highlight the fact that was observed in the group treated with DMCM a significant increase in NG2^+^ cells. One possible explanation is that the decrease of neuronal GABAergic response promoted an increase in neuronal activity. It is well known that neuronal activity is an essential factor of OPCs proliferation and differentiation (e.g., Barres and Raff, 1993; Demerens et al., 1996; Gibson et al., 2014), and that in general a bidirectional communication system between neuronal activity and OPCs modulates positively the process of myelination (e.g., Nishiyama et al., 2009; Kato and Wake, 2019). Thus, increase in neuronal activity by DMCM administration might produce an increase of NG2^+^ cells population as it has been demonstrated in several models. However, DMCM treatment that increases the NG2^+^ cell population does not lead to an increase in mature OLs. One explanation for this might be the inhibitory action of DMCM on the oligodendroglial GABA_A_ receptor, thus, suggesting that GABA_A_ receptor activation was necessary for OLs maturation. Another possibility is that differentiation towards mature OLs in this group was not observed due to the time-window analyzed here, and prolonged experiments would be necessary. The multiple mechanisms affected by systemic administration of β-carbolines in whole animal experiments encourage us to explore other possibilities in future studies. For example, it is known that affecting neuronal and glia activity in a general way as DMCM could do would also influence the participation of many other factors, in addition to GABAergic signalling pathways, factors that are known to be involved in regulating cell proliferation and differentiation of precursor cells, such as growth factors and other important neurotransmitters like glutamatergic and purinergic signals (e.g., Wake et al., 2011; Gautier et al., 2015; Welsh and Kucenas, 2018). In part, this complex response caused by DMCM seems to reveal the disadvantage of using a non-specific substance in the regulation of the GABAergic system, which affects both neuronal and glial activity.

In summary, our results show that myelination decreased by EB injection in the DRICP model is recovered by oligodendroglial GABA_A_ receptor-positive modulators, such as the β-carbolines β-CCB and β-CCE, through a mechanism that increases the number of OPCs and mature OLs in the lesioned area. These findings may have significant implications for white matter brain disorders that have an important component of primary damage to OLs and myelin.

## Data availability statement

The raw data supporting the conclusions of this article will be made available by the authors, without undue reservation.

## Author contributions

AC-M: Conceptualization, Data curation, Formal analysis, Funding acquisition, Investigation, Methodology, Writing – original draft, Writing – review & editing. RPO: Data curation, Formal analysis, Investigation, Methodology, Writing – review & editing. EG: Data curation, Formal analysis, Methodology, Writing – review & editing. ROA: Conceptualization, Data curation, Formal analysis, Funding acquisition, Investigation, Methodology, Supervision, Writing – original draft, Writing – review & editing.

## Footnotes

^1^ http://www.mrtrix.org
